# A Convolutional Neural-Network-Based Training Model to Estimate Actual Distance of Persons in Continuous Images

**DOI:** 10.3390/s22155743

**Published:** 2022-08-01

**Authors:** Yu-Shiuan Tsai, Alvin V. Modales, Hung-Ta Lin

**Affiliations:** Department of Computer Science and Engineering, National Taiwan Ocean University, Keelung City 202, Taiwan; 20857003@mail.ntou.edu.tw (A.V.M.); 4074w003@mail.ntou.edu.tw (H.-T.L.)

**Keywords:** rotation, deep learning, human skeletons, OpenPose, occluded human images, UAV application

## Abstract

Distance and depth detection plays a crucial role in intelligent robotics. It enables drones to understand their working environment to avoid collisions and accidents immediately and is very important in various AI applications. Image-based distance detection usually relies on the correctness of geometric information. However, the geometric features will be lost when the object is rotated or the camera lens image is distorted. This study proposes a training model based on a convolutional neural network, which uses a single-lens camera to estimate humans’ distance in continuous images. We can partially restore depth information loss using built-in camera parameters that do not require additional correction. The normalized skeleton feature unit vector has the same characteristics as time series data and can be classified very well using a 1D convolutional neural network. According to our results, the accuracy for the occluded leg image is over 90% at 2 to 3 m, 80% to 90% at 4 m, and 70% at 5 to 6 m.

## 1. Introduction

Distance detection plays a significant role in intelligent robotics, enabling drones to understand their working environment to avoid collision accidents immediately. Therefore, real-time multiple obstacle detection systems using stereo vision laser scanning equipment were proposed to avoid barriers for unmanned vehicles [1]. In addition, the Internet of Things (IoT) combines various perception and analysis technologies to improve manufacturing efficiency and quality [2]. Moreover, simultaneous localization and mapping (SLAM) [3,4] and a single-lens full mirror depth prediction model have been proposed [5]. Therefore, it can be seen that distance detection is essential in various artificial intelligence applications.

On the other hand, with the development of deep learning, the earlier image recognition algorithms have been impacted [6,7]. This resulted in a higher recognition quantity, a higher recognition accuracy rate, a high error-tolerant rate, and more diversified and rapid intention anticipation. With unmanned aerial vehicle (UAV) and automatic navigation technologies, UAV navigation decisions are usually based on image recognition combined with the integrated information of various sensors. Other applications calculate distance and self-relative orientation based on geometric information about objects. However, concerning human body position calculation [8], the geometric information in images is affected by the different clothes, postures, and physiques of human bodies. Furthermore, the sensor accuracy can be affected by the material of clothes. Therefore, these factors make estimating the body orientation and distance more challenging.

Consequently, there has been critical research emphasis on how to calculate the human body distance effectively. Large-scale enterprises or civil research institutes have provided numerous databases in recent years. They feature high-quality data and images with many objects [9,10,11]. Moreover, the images provided by Carnegie Mellon University consist of datasets of images at various angles, in-depth camera data, and rich sources of data, significantly affecting the development of deep learning. For example, in OpenPose [12], CMU human body image data were used as the training set, and the human body joints are taken as the training marks. Finally, The Residual Neural Network (RNN) predicts the human skeleton point, provides relatively high accuracy, and offers rich feature data for human orientation and distance evaluation.

In this study, we propose a method that can be used to estimate the distance of a human image when an object occludes the front of the image. First, we compute the feature points of the human image and then find the relationship between the human and the real-world coordinates of the camera projection. In addition, we recover the geometric errors caused by the different viewing angles of the human images to correct the actual distances of the human images in the camera images. The article is organized as follows: In Section 1, we introduce the current methods for distance estimation of human images, Section 2 introduces the related works, Section 3 introduces the experimental subjects and methods, Section 4 introduces our experimental procedure, and finally, we conduct the distance estimation test of the occluded human images. Finally, in Section 5, we give our conclusions and contributions.

## 2. Related Works

Relevant studies on the prediction of the human body turning [13,14,15] have been carried out, and their development trend can be divided into the following stages as follows. For example, one must extract the target image features with the histogram of oriented gradients (HOG) [16] of the images, count the histogram with the image edge features, and classify the shapes of the target image data with the artificial neural network. In regards to the database, the 3.6M Human dataset, which consists of more than 3.6 million 3D image training samples, is used. Then, the convolutional neural network (CNN) is trained using a convolutional neural network. Alternatively, the target image’s features are extracted from a public database, TUD [17].

Furthermore, one must intercept and miniaturize the person images and train with CNN [18]. Moreover, one must use the neural network model, residual neural network (ResNet) [19] with a high abstract image expression ability. On the other hand, High-Resolution Net (HRNet) [20] and the images with rich three-dimensional information use mixed open datasets and redefined tags.

Non-image sensors are one solution that can be used to determine the target’s distance by changing the frequency of light or sound. The distance can be calculated using the received reflection frequency for precise calculation, for example, infrared, ultrasonic, or optical radar (LIDAR). Typical applications are infrared cameras, robot navigation systems, self-driving assistance, road speed devices, and Kinect [21] physical game equipment. These devices have higher detection accuracy, are usually used for specific needs, and are more expensive. The other solution is to simulate the function of the human eyes. These methods use the object’s specific observation point to capture images at two different points in time and observe the spatial variation of parallax as a basis for judgment, usually using two cameras with different observation angles to calculate the estimated distance using Triangulation [22]. A typical application is the depth-of-field assisted prediction of smartphones. However, the accuracy of this method for accurate distance detection is limited, and if it relies on more than two lenses, the charge-coupled device (CCD) of the camera must have the same process to ensure that the internal parameters of the camera cause no error.

In image recognition, results vary due to the possible changing poses of people and various lighting. Illumination changes make color-based features unreliable. Since light changes make skeleton features inaccurate, Yu et al. propose a human re-identification method based on deep learning techniques with skeleton detection that can handle strong pose and light changes [23]. Zhao and Lu proposed a Neighbor Similarity and Soft-label Adaptation (NSSA) algorithm. The authors introduce a distance metric on the target domain that incorporates intra-domain neighbor similarity and inter-domain label adaptation regions [24]. Based on autoencoder architecture, Shi et al. proposed an unsupervised keypoint detector called Skeleton Merger [25]. The authors claimed Skeleton Merger could detect semantically rich and neatly aligned salient key points. However, the human body is non-rigid compared to a rigid body, so the recognition of the joints is more easily affected by clothing. In this study, we conducted a distance estimation experiment using the superiority of OpenPose for skeleton recognition. Therefore, we also want to test the effect of clothing on recognition in this study.

## 3. Materials and Methods

The primary implementation in this paper is for recognizing person images and involves the algorithm for estimating the distance between the person and the single-lens reflex camera. The research process can be divided into six items. First, we sampled the persons with single-lens reflex cameras and used the pre-training model OpenPose for the person’s skeleton point defection. Second, for image pre-processing, we referred to the camera position estimation method proposed by Kneip et al. [26] and calculated the changes in the camera position in the second image based on the feature points of the three fixed objects in the second image. The amount of change in the rotation angle of the camera can be calculated if its position is fixed, the object was rotated, and the feature points of the object were unchanged and detectable. We used the person framework coordinate point as the feature input of our artificial neural network because the amount of change in the rotation angle of the person could be calculated if the camera position was fixed and the persons in each of the images had the same features. Third, to estimate the person distance, we calculated the distance with the traditional lens distance measurement method, the focal length, and the pre-estimated actual shoulder width. In addition, we referred to the deep learning method of Turker et al. [27] and adopted the one-dimensional convolutional neural network as the primary classification method. Due to the classification of the persons’ rotation angles, the training and testing are carried out using a one-dimensional convolutional neural network to predict the actual rotation angles of the persons. The pre-treated skeleton data are featured by the one-dimensional data with a similar sound or semantic analysis [28]. Finally, the shoulder widths before the rotation of the persons are output based on the predicted angles and trigonometric function. Based on our model, we can restore part of the depth information lost due to person rotation.

**Figure 1:**
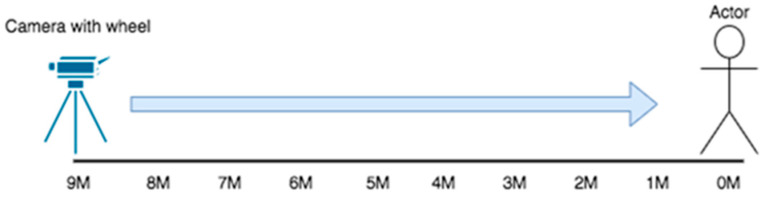
The camera tripod with pulley slowly moves from 9 m to 1 m before the subject.

**Figure 2:**
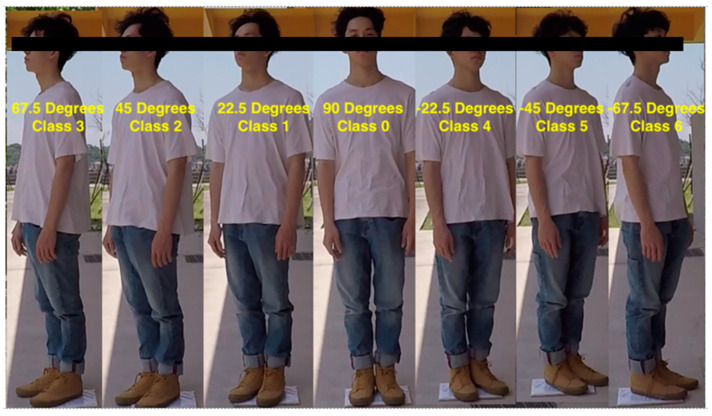
Image data with different rotation angles. (The eyes are marked for privacy consideration).

The recording started from 9 m and was slowly moved to 1 m before the person, as shown in Figure 1. Considering the completeness of the sampling data, we expected the manifestation of the persons that stand at the distance from 9 m to 1 m at the equivalent angle and the distance interval within 9 m. Therefore, all the distances are part of the training sample. For sampling quality consideration, the images used as the training sample were recorded at 60 frames per second (FPS60) with a resolution of 1080 pixels per inch (P1080). Therefore, the image data are constructed in Figure 2.

The linear photography function is mainly used to process the output images in advance through the chip in the camera. Thus, undistorted images can be obtained directly by eliminating the image distortions caused by lens surface curvature and the distortion of distorted images. The traditional distorted image processing method calculates the camera’s internal parameters with the checkerboard method [29]. Then, the calculated camera parameters are applied mechanically to each image. Thus, the function helps reduce the temporal and spatial complexity of the experiment.

**Figure 3:**
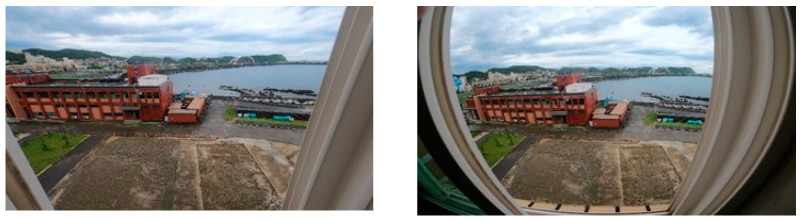
(**Left**) Non-distorted image; (**Right**) distorted image.

The estimation accuracy can be improved to a certain extent if the image distortion (Figure 3) is brought down to the lowest level. The distance estimation algorithm for images depends on the ratio between the number of pixels and the actual unit length in the vectors.

Regarding the data sampling part, the accuracy of the deep learning method is proportional to the data consistency of the training materials. The data consistency usually depends on the correctness of the data tags. A significant workforce is generally needed regarding tagging the training materials, and artificial tagging may be incorrect. The error rate of artificial tagging should still be considered in the experiment because the tagging mode was angle estimation, a personal judgment. Therefore, we applied the pinhole camera imaging principle to avoid a too high error rate of data tagging. Specifically, to ensure the consistency of the training materials and convenience in the implementation, we retained the capture position of the persons at the central imaging point of the camera (cx,cy). Expressly, *P* indicates the pixel of the person, and *C* is the central imaging point of the camera.

(1) ‖P−C‖≈0

**Figure 4:**
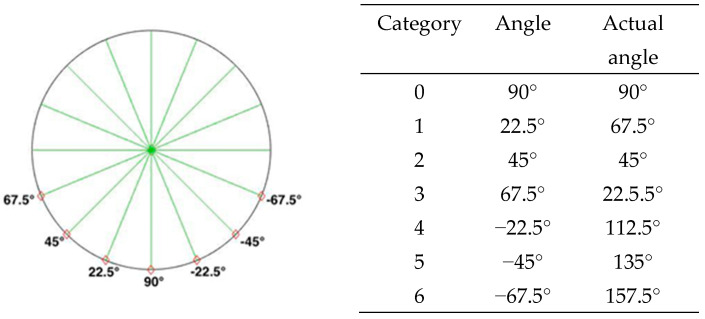
The rotation angle and classification method of the subject.

Regarding the image classification in the experiment, the influences of person rotation on distance estimation were emphasized. Therefore, the image classification was improved based on the method used by Yu [30]. First, we divided the person’s front side into seven rotation angles and tagged the data about the image of each of the angles (Figure 4). Then, we arranged the persons to be detected so they were aligned at the corresponding angle with an angle to minimize personal judgment systematically. 

First, we used OpenPose for our skeleton detection. It has an efficient human body posture detecting method that can transform persons’ limbs and torsos into feature points with geometric meanings. In the training material construction stage, we computed the skeleton points of each image and saved them in a JSON file. Each of the files contains the following information: (1) the total number of persons detected; (2) the skeleton points detected and undetected for each of the persons; (3) the pixel position, coordinates *x* and *y,* and confidence weight value of each of the skeleton points.

We fixed the persons to be detected at the central imaging point during the data sampling because multiple persons might synchronously detect the same image. The terms are listed as follows: $\vec{shd^{RL}}$ is the shoulder width vector; $shd^{R}$ is the coordinate point of the right shoulder; $shd^{L}$ is the coordinate point of the left shoulder; $shd^{M}$ is the coordinate point of shoulder width midpoint; $hip^{R}\;$ is the coordinate point of the right hip joint; $hip^{L}$ is the coordinate point of the left hip joint; $\vec{hip^{RL}}$ is the hip joint vector; $knee^{R}$ is the coordinates of the right knee; $knee^{L}$ is the coordinates of the left knee; $\vec{hip^{L}knee^{L}}$ is the vector of the left thigh; $\vec{hip^{R}knee^{R}}$ is the vector of the right thigh; $\vec{shd^{L}hip^{L}}$ is the vector of the left torso; $\vec{shd^{R}hip^{R}}$ is the vector of the right torso. Specifically, $P_{shd^{M}}$ is the shoulder width central point of the chosen subject. The term $p_{i}\;$ is the closest point to the imaging center point *C* among all candidates $p_{i\;shd^{M}}$. Moreover, n is all the subject skeleton points detected from the image.

To accomplish this, we used the advantage of an OpenPose [14] algorithm. In addition, we performed preliminary training on the model so that it can serve as one of the additional tools for our skeletal detection. As a result, it has an effective human body posture detection method that can turn subjects’ limbs and torsos into feature points with geometric meanings and can resist noise well. As a result, it will not be impacted by various noises of the subjects, including body shape, height, race, clothes, and other factors. 

**Figure 5:**
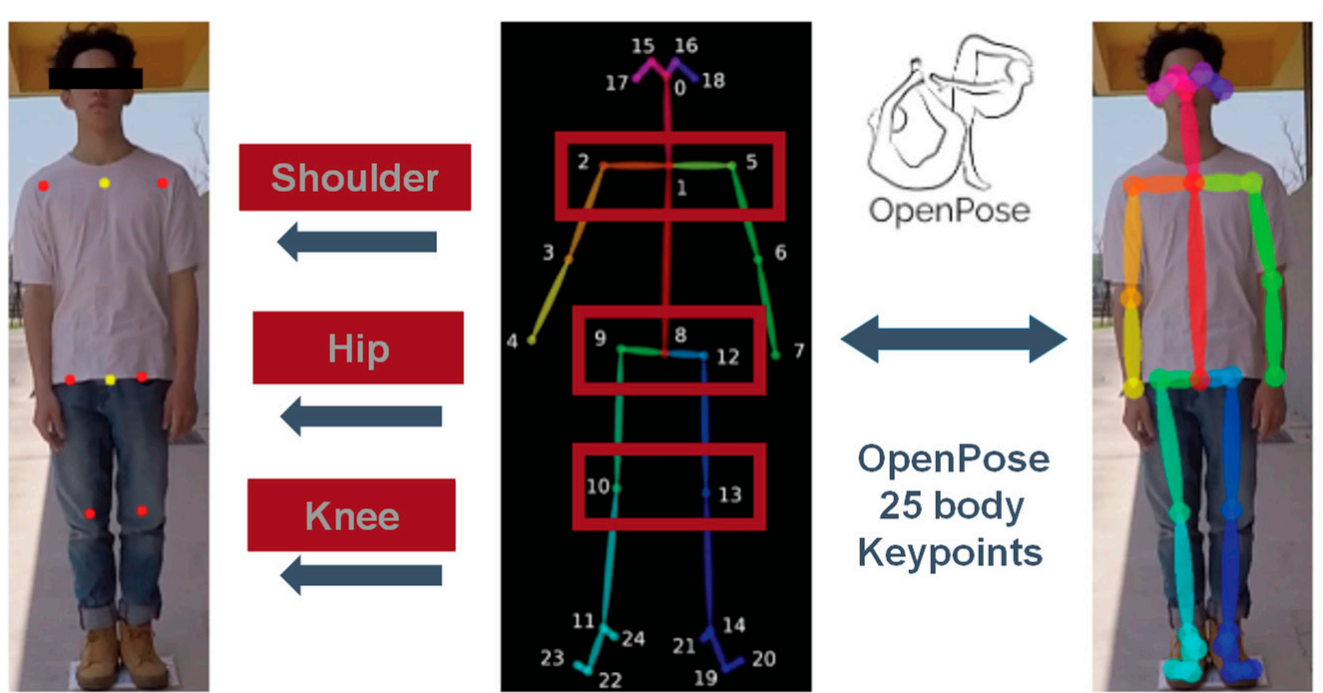
The correspondence table of OpenPose skeleton points.

During the training construction phase, we extracted the skeleton points of each image and saved them in a JSON file (4). Each of the files contains the following information: (1) the total number of subjects detected; (2) the skeleton points detected and undetected for each of the subjects; (3) the pixel position, coordinates x and y, and confidence weight value of each of the skeleton points (Figure 5).

We fixed the subjects to be detected at the central imaging point during data sampling because multiple subjects might synchronously detect the same image. Therefore, we could use Equation (2) to search for the subject nearest to the imaging center. The cross-reference for joint points is shown in Table 1. Specifically, $P_{shd^{M}}$ is the shoulder width central point of the chosen subject, $p_{i\;}\;$ is $p_{i\;shd^{M}}$ which is closest to the imaging central point *C* and is chosen to be the training sample from the candidate subjects, n is all the subject skeleton points detected from the image.

(2) $$P_{shd^{M}}=\underset{1\leq i\leq n}{\min}\|p_{i\;shd^{M}}-C\|$$

HOG was mainly used to draw the statistical histograms intuitively in which image gradient values were obtained from the images at various angles. Then, the decision tree or SVM was used to classify the target images. However, the existing person angle detection method is insensitive when the angles of the persons change slightly. Subsequently, with regards to the application of the deep learning method for the issue of predicting the 360 angles of rotation in eight directions, Choi and Lee [8] obtained an accuracy rate of about 80% based on an error tolerance scope of ±45 degrees with the CNN method and Human 3.6 M database.

However, the smaller the angle required for distance estimation, the better. According to Tsai et al. [31], we knew that any change in the pixel of the shoulder width would cause the distance to be distorted when the person tilts or rotates if the optical method is used to detect and measure the actual distance of the person. Therefore, the depth of the target object that we can restore can be more accurate if our system can identify a more delicate angle.

- Define human bodies’ geometric features

Regarding studying human bodies’ geometric features, we referred to Abidi [32], a study on the resolution of camera position. The amount of physical change of the quadrangle area in the target image varied with the shooting angle. Therefore, the camera position was reckoned to resolve the issue of camera position by using the geometric information generated by the amount of change. Consequently, we knew that we could take linearly divisible feature information to some degree by using the quadrangle formed by the left and right shoulders and left and right hips of human bodies and the amount of area change of the quadrangle.

Thus, regarding the features of the human skeleton points in the group, we used the mass center point of the quadrangle area as humans’ spatial coordinate axis of the coordinate system in the real world. We used geometric features in order to use the relatively fixed features in each image as the normalized datum values to bring to prominence the vastly changing part.

(3) $$A^{L}\Delta =\frac{\vec{shd^{R}hip^{L}}+\vec{shd^{L}hip^{R}}+\vec{shd^{L}hip^{L}}}{2}\;A^{R}\Delta =\frac{\vec{shd^{L}hip^{R}}+\vec{shd^{L}hip^{R}}+\vec{shd^{R}hip^{R}}}{2}$$

Moreover, for the skeleton coordinates for each image, we adopted the area calculation method provided by Heron’s Equation (3). As the pre-processing of the center coordinates of the person’s torso, the areas formed by the torso were firstly calculated. Specifically, $A^{L}$ and $A^{R}$ indicate the left and right parts of the torso divided into two halves by the left shoulder and right hip pixels.

(4) $$C_{L}^{x}=\frac{shd_{L}^{x}+hip_{L}^{x}+hip_{R}^{x}}{3}\;C_{L}^{y}=\frac{shd_{L}^{y}+hip_{L}^{y}+hip_{R}^{y}}{3}$$

To obtain the quadrangular center coordinates of the torsos, we needed to obtain the rectangular coordinate system of the triangular mass center of $A^{L}\Delta$ and $A^{R}\Delta \;$ in advance, respectively, and we could obtain this through Equation (4): $A^{LR}\Delta$ is at the triangular center coordinates $C_{L}=\left(C_{L}^{x},C_{L}^{y}\right)$ and $C_{R}=\left(C_{R}^{x},C_{R}^{y}\right)$. We could calculate the central rectangular coordinate system formed by the torsos with Equation (5). Specifically, $C_{body}^{x}\;$ is the coordinates of axis *x* of the central point of the torso, and $C_{body}^{y}$ is the coordinates of axis *y* of the central point of the torso. Referring to the results of Equations (3) and (4), they can be described as:

(5) $$C_{body}^{x}=\frac{C_{L}^{x}*A^{L}+C_{R}^{x}*A^{R}}{A^{R}+A^{L}}\;C_{body}^{y}=\frac{C_{L}^{y}*A^{L}+C_{R}^{y}*A^{R}}{A^{R}+A^{L}}$$

The center coordinate value $C_{body}.$ The torso can be obtained based on Equation (6).

(6) $$C_{body}=\left(C_{body}^{x},C_{body}^{y}\right)$$

We define *P* as the OpenPose feature points through $\vec{shd^{R}hip^{R}}$, $\vec{shd^{R}shd^{L}},$ $\vec{shd^{L}hip^{L}},$ $\vec{hip^{R}hip^{L},}$ $\vec{hip^{L}knee^{L},}$ and $\vec{hip^{R}knee^{R}}$.$\;P_{i}$ denotes the different parts of *P*, and we take *j* equidistant points from each side of $P_{i}$ to form different coordinates *v*. We then calculate the unit vector of the torso based on Equation (7). The quadrangular central point $C_{body}$ and arithmetic distance coordinates $\;P_{ij}$ of the torso normalized the joints that we were interested in and obtained the unit vector coordinates $\vec{u_{ij}}$, the feature data of the skeleton point of the person, and took it as the input item ψ of the artificial neural network.

(7) $$\psi \in \left\{\vec{u_{ij}}\right\}=\frac{P_{ij}-C_{body}}{\left\|P_{ij}-C_{body}\right\|}$$

- Construct rotation angle classifier

In most cases, it is impossible to know the angle at which the person in an image or photo faces the lens. In real life, the azimuth the person faces cannot wholly coincide with the estimated amount in the image. To quantify the angle of each of the photos as far as possible, we classified the predicted angles of the person into seven categories, which were at an interval of 22.5 degrees, as shown in Equation (8). Specifically, $O^{P}$ is the possibility that the person is at a rotation angle.

(8) $$O^{P}=\left\{p_{i}|i=\theta_{class1},\theta_{class2},\theta_{class3},\theta_{class4},\theta_{class5},\theta_{class6},\theta_{class7}\right\}$$

As above, multiple classifiers as the input data provide the feature vectors of the persons. The weighted values of multiple classifiers were updated with the mean squared error loss function to calculate the probability value of the classification results. It can be expressed from the relation of Equation (9) as:

(9) $$O^{P}=\max\left(p_{i}\right)$$

The term $O^{P}$ denotes that the maximum value should prevail. Therefore, predicting the angles of the persons fell into a category.

We chose to estimate the angles of FPS60 continuous images to enable angle estimation in continuous images. Then, we sampled and calculated the predicted results of every five images at an interval of 2, obtained their average direction, updated our rendering information with OpenCV, and established visual evaluation results.

Equation (10) describes the method to predict the angles of continuous images. $I_{k}$ is each of the image frames; we took out the results of all the numbers with the unit estimated value of every *n* images, and rendered their estimated values on the persons $J_{t}\left(O^{P}\right)$ in the image. Specifically, t is the target number of rendered frames, $O^{P}$ is the estimated direction value of a person in each of the images.

(10) $$J_{t}\left(O^{P}\right)=mode_{n\left(t-1\right)<k\leq nt}\left\{I_{k}\left(O^{P}\right)\right\}$$

We used a four-layer one-dimensional convolutional neural network with a ReLU excitation function to classify the feature vectors, and the neural network architecture is illustrated in PyTorch symtax as follow: 

self.convnet = nn.Sequential(  nn.Conv1d(in_channels = initial_num_channels, out_channels = 512, kernel_size = 3, stride = 2),  nn.ReLU(),  nn.Conv1d(in_channels = 512, out_channels = 128,kernel_size = 3, stride = 2),  nn.ReLU(),  nn.Dropout(0.2),  nn.Conv1d(in_channels = 256,out_channels = 64, kernel_size = 3, stride = 2),  nn.ReLU(),  torch.nn.Dropout(0.2),  nn.Conv1d(in_channels = 256, out_channels = 32, kernel_size = 1, stride = 1),  nn.ReLU()  nn.Conv1d(in_channels = 64,out_channels = 32, kernel_size = 1,stride = 1),  nn.ReLU() ) self.fc = nn.Linear(64, num_classes)

- Real-time multiple persons rendering method for continuous images

The visual image rendering method was used to verify the practical degree of the model. After the artificial neural network model was trained and tested, the verification data were treated. After their features were extracted, the verification data were continuous images and were input into our artificial neural network. Then, the angles of rotation of the persons in the images were directly predicted.

Then, the verification process can take out all the joint point information of the human bodies in each image with the API provided by OpenPose. In this way, the joint points we are interested in can be operated; we can extract their feature vectors and treat every five images as one batch of recognition units (batch size = 5). The feature vectors of 5 images indicated that it had already been obtained when the set matrix length was up to 5, and 5 predicted values could be obtained after the feature vectors were input into our trained model.

We rendered all the persons on each image with the Equation (10) voting method. However, we used the neck joints of the persons as the initial coordinates. The sequences of skeleton points of the persons can vary with the number of persons in each image as the lens moves. Moreover, the nearest neck joints were compared among every five images to ensure that the same candidate person was involved in every five images when the voted person turned.

We calculated the shoulder width proportion required to be restored when the person faces a certain degree, as shown in Table 2. Under the camera imaging principle, the data about axes *x* and *y* proportional to the coordinates in the real world were retained for the persons in the images. The *x* coordinate element was used after the rotation of the persons. According to Equation (11), $\vec{shdX^{RL}}$ is the x-axis element of the rotated shoulder width, arccos(θ) is the corresponding arccos anti-trigonometric function coefficient, and $\vec{shdX_{orig}^{LR}}$ is the x-axis element value of the restored shoulder width of the person. The actual distance could be restored under the camera imaging principle.

(11) $$\vec{shdX_{orig}^{LR}}=\vec{shdX^{RL}}/cos\left(\theta \right)$$

(12) $$l=\frac{f*shd_{real}}{\vec{shdX_{orig}^{LR}}}$$

We assumed that the actual shoulder width of 35 cm is the average shoulder width of adults, *d* is the number of shoulder width pixels in the images, *f* is the focal length, and *l* is the actual distance between the target object and the pinhole. Equation (12) is the calculation method for the actual distance of the persons. Specifically, *f* is the actual distance between the person and the camera, $shd_{real}$ is the actual shoulder width, *f* is the focal length, $\vec{shdX_{orig}^{LR}}$ is the distance between the x-coordinates of the two shoulder image pixels.

## 4. Results and Discussion

Twenty persons were used for the training samples. A classification test was carried out for the angles of the persons with the rotation angle classifier. The test samples were taken based on nine distance levels, i.e., 1 m, 2 m, 3 m, 4 m, 5 m, 6 m, 7 m, 8 m, and 9 m. The training samples were constructed gradually from 9 m to 1 m. The camera approached the person at a speed of 1 m per 5 s to record in 60FPS with a resolution of 1080P.

- Critical distance test for classifier

At a 1 m distance, the knee skeleton point could not be judged and read since the distance was too close. As for the skeleton rendering method in the OpenPose, the right and left shoulders were defined as green and orange, respectively. Therefore, the rendering within 6 m was regarded as standard. However, at a distance of 9 m, there is a certain probability that the left and right shoulders feature points are recognized as abnormal because the image will be too vague under a resolution of 1080 P. In addition, the left and right shoulders were exchanged, resulting in apparent noise compared with the data samples within 6 m.

The left and right shoulders and coordinates were standard at 8 m. Similarly, the hip joint at a distance of 9 m was abnormal because the hip joint manifested instability. Thus, a great mistake happened when the rotation angles were classified, the left and right shoulders were exchanged, and the hip joint was abnormal.

**Figure 6:**
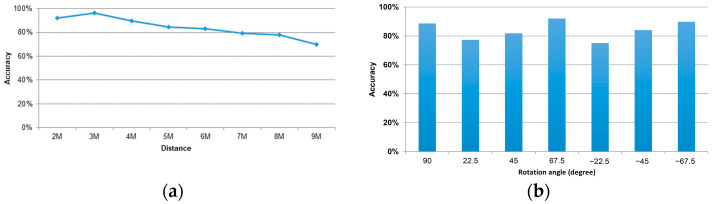
The average accuracy rate of 11 persons is indicated based (**a**) on distances; (**b**) on rotation angle in degree.

According to the statistics shown in Figure 6, training samples of 20 people were used. Therefore, 20% of the data were used as test samples for the artificial neural network. All persons’ distances were tested with the neural network weight under a convergent state. Specifically, the x-axis is the distance from 2 m to 9 m, and the y-axis is the average test accuracy for the 11 persons. The accuracy calculation method was as follows: One must consider that the success rate of the neural network outputs in seven directions could be wrong at each distance because the person faces the camera at an angle of 90 degrees. Therefore, it was similar to the categories of +22.5 and −22.5 degrees, and we could adopt these categories. The reason may be that the recognition might fail, and 22.5 degrees was liable to be read as 90 degrees under the influence of body shape and clothes when the angle was slight.

In the experiment, we found that most persons’ knee skeleton points at a distance of 1 m were undetectable, resulting in low resolution. Thus, they were not included in the calculation. The results between the interval of 2 m and 3 m were the best, with an average accuracy rate of about 90%, as the skeleton point was clear and the noise and interference were small. At a distance of 3 m to 4 m, the average accuracy rate reached more than 80%. It slowly increased from 80% to nearly 70% at the distance interval of 5 m to 7 m, and it was less than 80% at a distance of 8 m to 9 m due to the resolution limitation.

- Actual distance estimation test

The distance between the persons and the camera was restored and calculated with the persons’ left and right shoulder coordinates. The focal length of the camera and the average shoulder width of human bodies are shown in Equation (11). Due to the varying angles of the individual’s face, this procedure is divided into two actual examples. First, there was no distortion due to the maximum shoulder width in this case. Second, we considered the angles at which the person’s face changes. In this case, the shoulder width is changed because the angle the person faces is 67.5 degrees; accordingly, distortion happened when the absolute distance was calculated.

We corrected the shoulder width distances of three distortion cases, 22.5, 45, and 67.7 degrees, with Equation (11). We corrected the three distortion conditions with the artificial neural network model to restore the pixel distance distortion after person rotation as far as possible. The corrected shoulder width would be evaluated based on the shoulder width distance at which the persons face the camera. The test method is as follows: within 2 to 9 m of the 11 persons, one must evaluate the seven angles every 1 m. We discuss the difference between the situations under which the rotation angle classifier was not used in two parts.

- Evaluation of the effectiveness of shoulder

**Figure 7:**
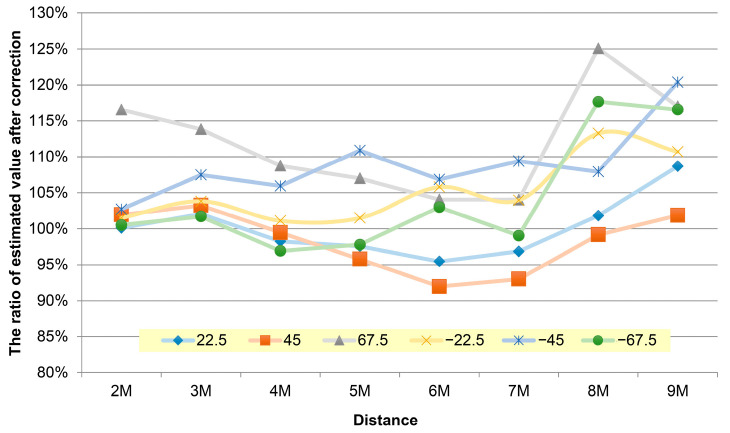
The average broken line graph of the supplemented and corrected error values of the shoulder width coordinate distance of the persons in the images at various angles and within 2 m to 9 m.

**Figure 8:**
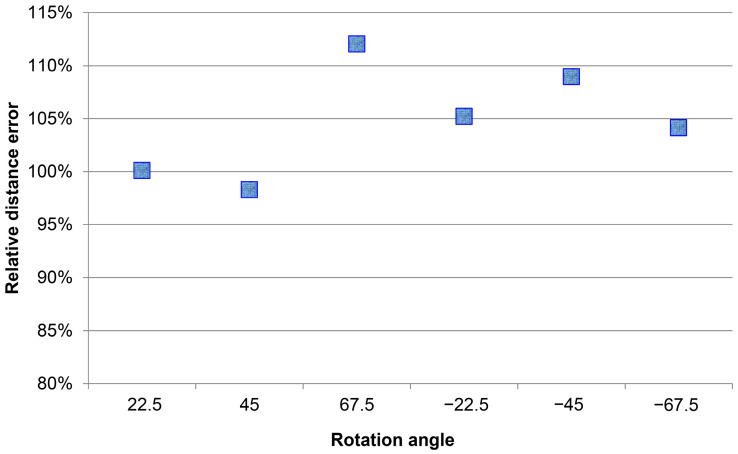
Average correction value of face angle.

**Figure 9:**
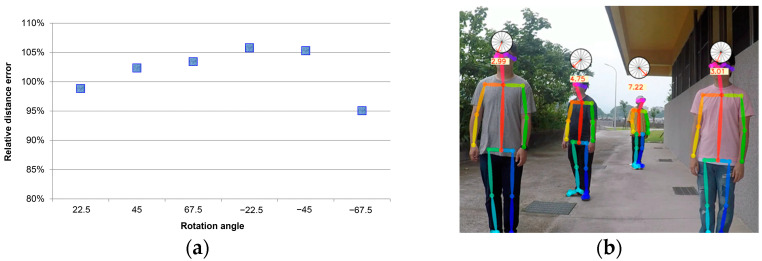
(**a**) Relative error of the estimated distance using angle correction. (**b**) Estimation of actual distance using rotation angle.

A significant error was generated due to the relationship between the unstable left and right shoulder coordinates, except for the significantly changed 67.5 and −67.5 degrees. As the distance increased, a large angle tended to cause the difference between the corrected shoulder width and the front shoulder width, and most of the test samples were within the interval of ±20%. The y-values in Figure 7, Figure 8 and Figure 9 represent the result of using the angle estimation, returning the shoulder width to the frontal image, and calculating the distance.

Figure 7 shows the restoration of the average shoulder width at each distance. Under the worst condition, more than 16% of the 67.5 degree shoulder width in the distance of 9 m was corrected and restored. More than 19% of the −45 degree shoulder width in the distance of 9 m was restored, except at the large angles. Significant errors could be caused due to the correction of the shoulder coordinates when the visual angle was too broad.

In Figure 8, where the restoration proportion was calculated on average in angle categories, the results were ideal, except that the restoration proportion at 67.5 degrees was more than 11.5%. This signaled that restoring the shoulder width with angles had a significant effect in most cases.

- Evaluation of the effect of rotation angle classifier

The predicted result was included in the corresponding cos(θ)  value. The corrected error value was calculated with the undistorted shoulder width, and at some of the data points like 45 degrees, a significant error was caused due to wrong category estimation. Large errors were generated because the supplemented and corrected shoulder widths were partially overlapped or the left and right shoulders were exchanged and were out of position, thus failing to express the geometric features. The correction points were generated because the shoulder widths could not be easily detected when each person rotated except at an angle of ±67.5 degrees. The data points outside ±67.5 degrees mostly moved towards an accuracy of 100%. We could effectively evaluate the angles the person faces by rendering the angles in categories based on Equation (10). The average accuracy of each of the distances after the estimated values of the person’s directions was mechanically applied. Besides the accuracies in all directions after 8 m and at an angle of ±67.5 degrees, which had a significant average error, it was within a 10% error range.

As shown in Figure 9a, the relative error of the estimated distance using angle correction applied directions of the persons indicated that the shoulder width to be restored at the average correction distance would only have an error of ±5%. After the corresponding correction, value cos(θ) was chosen for each of the directions.

It had 100% recognition accuracy at 2 m, 3 m, and 4 m. The persons in a single color had an excellent recognition accuracy rate at 2 m and it was unstable and even less than the average value at 3 m and 4 m. At a 5 m distance, the seven angles were recognized entirely, which was considered an exception. After 5 m, the average accuracy rate tends to decrease. After 5 m, the classifier data were exposed to noise and interference. As the distance increased, the accuracy rate reduced, and it was approximate to the average value, although exceptions happened in some of the test samples after 5 m. In particular, the data for persons with serious interference were more liable to have an abnormal accuracy rate.

- Occluding influence test

**Figure 10:**
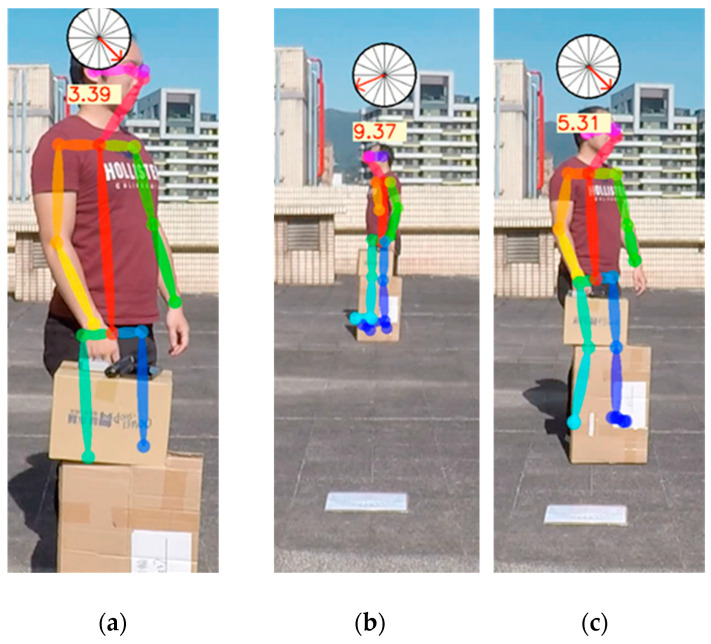
Occluding below the knee; (**a**) 3 m, (**b**) 9 m, and (**c**) 5 m.

The occluding image test is shown in Figure 10. In most cases, masking could cause some feature points to disappear; for example, the ankle joint could not be distinguished [33,34]. However, the predicted angle value could be correctly output in most cases. Consequently, the influences on direction prediction were minor because we did not refer to the joint points below the knees for extracting our feature points when the persons were partially occluded.

**Figure 11:**
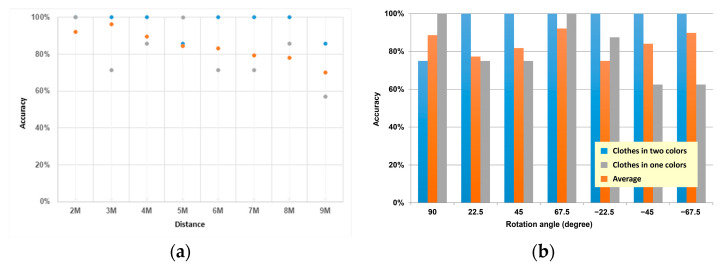
Comparison of distance accuracy of clothing differences for (**a**) distance and (**b**) rotation angle.

Since skeleton detection, such as OpenPose, may be influenced by the human image, it may be more challenging to distinguish the single-color pattern farther away, so we conducted the test for the clothing color. We compared the difference between single-color clothes and two-color clothes at different distances and rotation angles. It was found that the recognition accuracy was better in two-color clothing, with 100% recognition accuracy at 2 m, 3 m, and 4 m (Figure 11a). In the case of monochromatic clothing, except for 2 m, the performance of 3 m and 4 m was unstable and even lower than the average accuracy rate. The fact that all seven angles were successfully identified at 5 m is only an exceptional case because the average accuracy rate after 5 m showed a decreasing trend, indicating that the data after 5 m show a situation with more serious noise interference for the classifier. The accuracy rate decreases significantly with increasing distance.

Nevertheless, the actual performance is still close to the average accuracy, especially for the data with more serious interference, which are more likely to have abnormal accuracy. For example, for the different rotation angles (Figure 11b), the performance was good for all angles in the two-color clothing, except for two errors at 5 m and 9 m at 90 degrees. In contrast, the error rate was significantly higher in monochromatic clothing. The reason may be that the prediction point of the hip relationship for monochromatic characters differed from that for bicolor characters.

## 5. Conclusions

In this study, we used a single lens to deal with the problem of large distance calculation error caused by the rotation. Based on the experimental data and results, we came to the following conclusions. (1) The unit vector of character skeleton points can be well classified in a one-dimensional convolutional neural network after normalization. (2) Training on the accuracy of data labeling can be used to determine if the human face orientation can be accurately predicted. (3) Performance is normal on known labels. However, there is room for improvement in the in-between category and category perspective. (4) Our method can provide direction and distance estimates for partially obscured images of people. Our study had some limitations. The number of experimental participants in this study was relatively small, but we compensated for this problem by exploiting the regularization of human skeletal points. In addition, this study uses OpenPose to recognize skeleton points. In the future, we will try different skeleton point recognition methods, such as autoencoder, and increase the number of trainees to improve this method’s recognition rate. Furthermore, if the same classifier is further designed for the depression angle of the image, it can be effectively applied to the unmanned aerial vehicle to improve its control safety. 

## Figures and Tables

**Table 1:** Parameter corresponding to OpenPose points.

Mark	Note
$\vec{shd^{RL}}$	Shoulder width vector
$shd^{R}$	Coordinate point of right shoulder
$shd^{L}$	Coordinate point of left shoulder
$shd^{M}$	Coordinates of the shoulder width midpoint
$hip^{R}$	Coordinates of the right hip joint
$hip^{L}$	Coordinates of the left hip joint
$\vec{hip^{RL}}$	Hip joint vector
$knee^{R}$	Coordinates of the right knee
$knee^{L}$	Coordinates of the left knee
$\vec{hip^{L}knee^{L}}$	Vector of left thigh
$\vec{hip^{R}knee^{R}}$	Vector of the right thigh
$\vec{shd^{L}hip^{L}}$	Vector of the left torso
$\vec{shd^{R}hip^{R}}$	Vector of the right torso

**Table 2:** Comparison of trigonometric functions.

Facing Degree	Shoulder Angle	cos(θ)
90	0	1
22.5 or −22.5	67.5 or −67.5	0.9239
45 or −45	45 or −45	0.7071
67.5 or −67.5	22.5 or −22.5	0.3827

## Data Availability

Not applicable.
